# Effect of laser acupuncture on dry eye: A study protocol for a 2-center randomized controlled trial

**DOI:** 10.1097/MD.0000000000010875

**Published:** 2018-06-01

**Authors:** Wen-Long Hu, Pei-Chang Wu, Li-Yen Pan, Hun-Ju Yu, Chih-Chin Pan, Yu-Chiang Hung

**Affiliations:** a Department of Chinese Medicine, Kaohsiung Chang Gung Memorial Hospital and Chang Gung University College of Medicine; b Fooyin University College of Nursing; c Kaohsiung Medical University College of Medicine; d Department of Ophthalmology, Kaohsiung Chang Gung Memorial Hospital and Chang Gung University College of Medicine, Kaohsiung; e Department of Ophthalmology, Linkou Chang Gung Memorial Hospital, Taoyuan; f Sunming Eye Clinic, Kaohsiung; g School of Chinese Medicine for Post Baccalaureate I-Shou University, Kaohsiung, Taiwan.

**Keywords:** dry eye, laser acupuncture, traditional Chinese medicine

## Abstract

**Background:**

Dry eye is a common ophthalmologic disorder that causes ocular discomfort and has become a worldwide health concern. Patients with dry eye often turn to complementary and alternative medicine (CAM) because of unsatisfactory conventional treatments. Acupuncture is one of the most popular interventions of CAM used, and laser acupuncture (LA) is a noninvasive technique.

**Methods:**

This protocol is a 2-center randomized controlled trial investigating the effect of laser acupuncture on dry eye. Two hundred participants aged 20 to 65 years will be randomly assigned to the experimental group (LA plus conventional treatment) or the sham control group (LA without laser output plus conventional treatment) at 2 clinical research centers in South Taiwan. The subjects will undergo LA treatment thrice a week for 12 weeks. The subjects in the experimental group will sequentially receive 0.375 J of energy at each of the following acupoints: BL2, TE23, ST2, LI4, ST36, and GB37. The subjects in the control group will also receive a sham LA treatment, without any laser output. Outcome assessments will include evaluation of the ocular surface disease index (OSDI), tear film break-up time (TFBUT), Schirmer-I test finding, and the visual analog scale (VAS) score at 4 and 12 weeks before treatment. The OSDI, TFBUT, Schirmer-I test result, and VAS score of the participants will be analyzed and compared between the experimental and control groups using the paired *t* test and one-way analysis of variance.

**Objectives:**

The aim of this protocol is to investigate the efficacy of LA therapy in patients with dry eye.

**Trial registration:**

ClinicalTrials.gov NCT03204903.

## 1 Introduction

Dry eye or the dysfunctional tear syndrome (DTS) is a multifactorial disease characterized by a poor production of tears, leading to a dry ocular surface. Symptoms of dry eye include visual disturbance and tear film instability with potential damage to the ocular surface. The 2 major causes of dry eye are aqueous deficiency and increased evaporation.^[1]^ The global prevalence of dry eye ranges from 5% to 35% and therefore, has become a worldwide health concern.^[1,2]^ It is more common in those aged >50 years and affects mainly women (70%), and causes significant loss of productivity at work, as there is no effective therapy.^[1]^

Diagnosis and management of DTS are often challenging and a source of frustration for eye care professionals and patients with this condition.^[3,4]^ Artificial tears, gels, ointments, and inserts are used to replenish the tear film.^[5]^ However, such treatments are often palliative and inadequate in providing satisfactory relief from debilitating symptoms.^[6]^ Patients with dry eye, therefore, often turn to complementary and alternative medicine (CAM). Acupuncture therapy is one of the most popular forms of CAM, which is used for the treatment of a variety of conditions.^[7]^ While some recent studies have suggested that acupuncture could be effective for dry eye,^[8–10]^ others have failed to reach a consensus regarding the most effective acupuncture treatment method, owing to low methodological quality and insufficient sample sizes and therapeutic sessions.^[11,12]^

Acupuncture is one of the most popular interventions of CAM used. Laser acupuncture (LA) is a noninvasive technique that involves stimulation of traditional acupoints with low-intensity, non-thermal laser irradiation. LA has been used for the treatment of several eye conditions including amblyopia.^[13,14]^ LA integrates the positive effects of both acupuncture and low-level laser therapy.^[15]^ The aim of this study is to investigate the effects of LA on dry eye, based on the traditional Chinese medicine (TCM) theory, which advocates treatment of the body acupoints to replenish the tear film.

## 2 Methods

### 2.1 Ethics approval

The study was approved by the Human Ethics Committee of our hospital (Chang Gung Medical Foundation Institutional Review Board, IRB No. 201600333A3). The protocol was registered with ClinicalTrials.gov (Identifier: NCT03204903). Written informed consent will be obtained from all of the participants. Personal information about potential and enrolled participants will be collected, shared, and maintained in an independent closet in order to protect confidentiality before, during, and after the trial.

### 2.2 Study design

**Figure 1:**
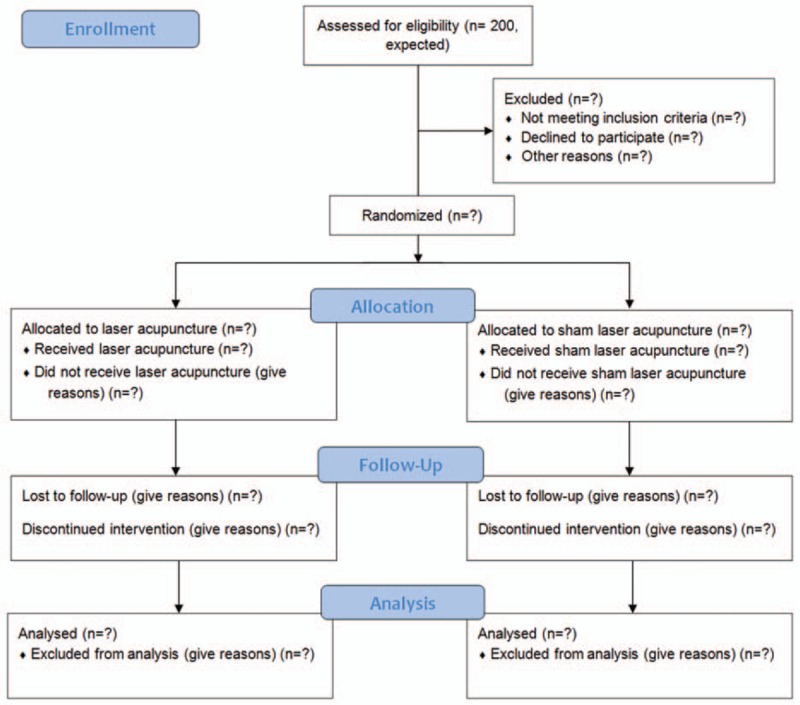
The flowchart of the trial.

This 2-center, randomized, controlled trial is being conducted at the Department of Ophthalmology, Chinese Medicine at the Kaohsiung Chang Gung Memorial Hospital and the Sunming Eye Clinic, 2 clinical research centers in South Taiwan from August 2016 and will continue until July 2018. The participants independently recruited by each center will be randomly allocated to the experimental group (LA plus conventional treatment, n = 100) or the sham control group (LA without laser output plus conventional treatment, n = 100). All participants will receive 36 sessions of treatment over a 12-week period. The study design is depicted in Fig. 1.

### 2.3 Participants

Diagnosis of dry eye is established on the basis of patient symptoms, assessed using the ocular surface disease index (OSDI) questionnaire, as well as visual disturbance (blurred vision or poor vision) or visual function (problems reading, driving at night, working on a computer, or watching TV) assessment; tear film break-up time (TFBUT) measurement and Schirmer test for assessing tear film stability; ocular surface staining; Optikgerate keratograph for assessing ocular/conjunctival redness; and interferometry using the LipiView II Ocular Surface Interferometer (TearScience, Inc., Morrisville, NC) device for assessing Meibomian gland dysfunction.

Patients aged 20 to 65 years with aggravating acute or chronic dry eye symptoms in one or both eyes were recruited. Physicians and ophthalmologists assessed the participants’ eligibility to participate in the study. The inclusion criteria were based on the following ophthalmologic test results: a TFBUT below 10 seconds and a Schirmer I test (with anesthesia) value below 5 mm/5 min. These tests were administered by ophthalmologists who had no information about the allocation results. Participants with eye lesions, diabetes mellitus, vitamin A deficiency, sequelae of Bell's palsy, a critical illness such as the Stevens–Johnson syndrome, cancer, infectious disease of the skin, a history of seizure or epilepsy; those who had undergone an eye operation in the past 3 months or had undergone hemodialysis; those using a combination of other ophthalmic medications, contact lens, a pacemaker, or immunosuppressants; those who had taken or needed active treatment (including Chinese medicine); and those who did not meet the physician's assessment for recruitment or was unwilling to provide informed consent were excluded. In addition, pregnant women were excluded.

### 2.4 Sample size and randomization

The necessary sample size was calculated from the results of a previous study on the effects of acupuncture. The mean difference (standard deviation) of the OSDI after acupuncture treatment was –17.61 (15.61), and after sham acupuncture, was –17.20 (18.81).^[11]^ Anticipating a 15% dropout rate, a total of 200 participants (http://powerandsamplesize.com) were recruited and evenly assigned to each center (100 participants in each center). Participants were randomly allocated to either an experimental or a sham control group using a research randomizer (https://www.randomizer.org). We equally randomized patients who were refractory to conventional treatment and those who were stable and responded to conventional treatment. The trial participants, outcome assessors, and data analysts will be blinded after assignment to interventions using label A and B for the 2 groups.

### 2.5 Interventions

There are different treatments for dry eye including topical medication and surgical intervention. In this study, we choose patients who only received artificial tears with or without a weak steroid, that is, 0.02% fluorometholone before or at the start of treatment at the beginning of the study. We will not change treatment medication until completion of study.

**Figure 2:**
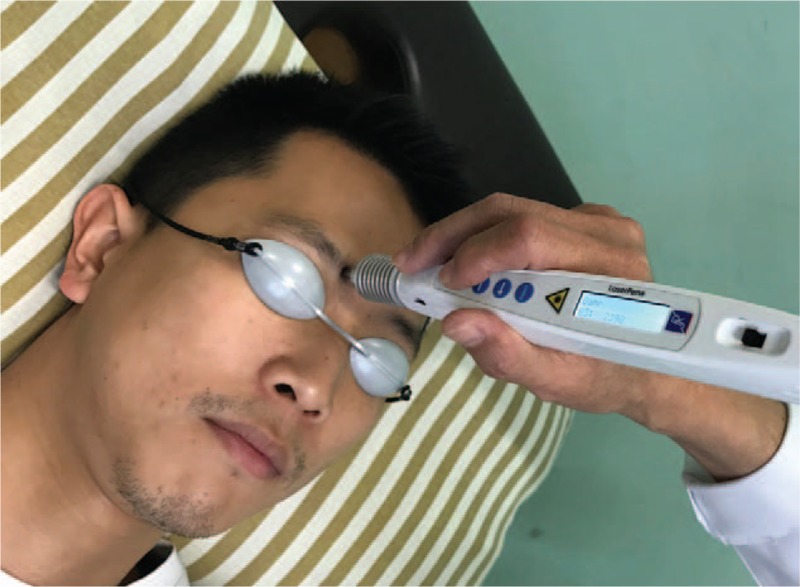
Laser acupuncture performed using the LaserPen device at the BL2 acupoint.

**Figure 3:**
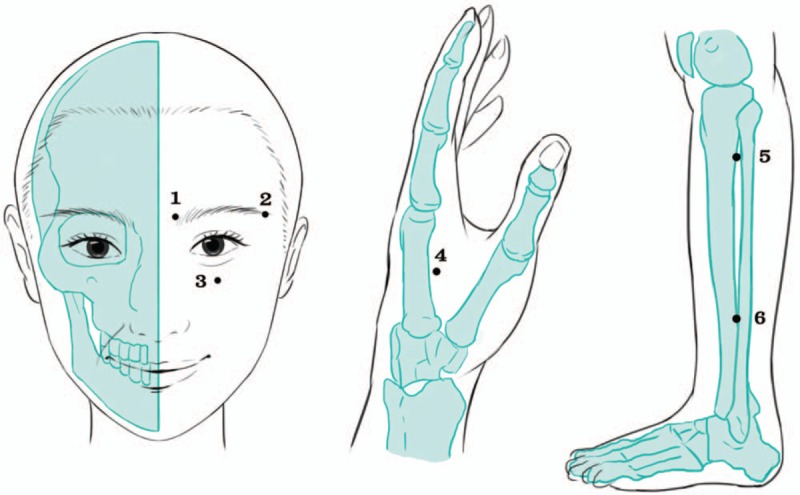
Acupoints used for dry eye: 1. BL2, 2. TE23, 3. ST2, 4. LI4, 5. ST36, and 6. GB37.

The subjects in each group will undergo 36 sessions of activated LA treatment, thrice a week, for 12 weeks, using a gallium aluminum arsenide LaserPen (maximal power, 150 mW; wavelength, 810 nm; area of probe, 0.03 cm^2^; power density, 5 W/cm^2^; pulsed wave; and Bahr frequencies [B1: 599.5 Hz, B2: 1199 Hz, B3: 2398 Hz, B4: 4776 Hz, B5: 9552 Hz, B6: 19,104 Hz, and B7: 38,208 Hz]; RJ-Laser, Reimers & Janssen GmbH, Waldkirch, Germany). We will use the same acupoints in both the experimental and the sham groups. The subjects in the control group will receive sham LA treatment, without any laser output (no stimulation), while the subjects in the experimental group will sequentially receive 0.375 J of energy at each of the following acupoints: BL2 (Cuanzhu, B3) (Fig. 2), TE23 (Sizhukong, B3), ST2 (Sibai, B3), LI4 (Hegu, B3), ST36 (Zusanli, B2), and GB37 (Guangming, B2) (Fig. 3). The laser treatment will be applied to each point for 5 seconds, to deliver a total treatment dose of 4.5 J/cm^2^. All acupoints are selected and localized according to the WHO Standardized Acupuncture Point Location guidelines.^[16]^ In all the subjects, the laser application will be performed by the same experienced physician who has sufficient training and is a licensed Chinese medicine practitioner in Taiwan. Protective goggle and laser shield were used by the operator and the patient to inhibit visual perception during LA, respectively.

### 2.6 Outcome measurements

Both eyes will be assessed for outcomes, 4 and 12 weeks after the first visit. Outcome measurements include 2 aspects, subjective ophthalmologic tests and objective questionnaires for ocular symptoms related to dry eyes. The primary outcome is the difference in the OSDI changes between the 2 groups. The secondary outcomes are the differences in TFBUT, Schirmer I test (with anesthesia) score, and 10-cm visual analog scale (VAS) score of eye discomfort.

OSDI is a 12-item scale for the assessment of symptoms related to dry eye disease and their effects on vision.^[17]^ The scores range from 0 to 100, and higher scores represent a more severe dry eye state. TFBUT measures the time required for tears to evaporate and diffuse after blinking naturally.^[18]^ A Schirmer I test (with anesthesia) is used to assess how quickly the eyes produce tears.^[19]^ A 10-cm VAS (0–10 points, least to greatest discomfort intensity) is used to measure the intensity of eye discomfort. The OSDI and VAS score are assessed by separate outcome assessors who did not perform the LA treatment. TFBUT and Schrmir I test outcomes are evaluated by ophthalmologists.

The reasons for patients not completing the follow-up visits or dropping out of the study, such as adverse events/intercurrent illnesses, suboptimal response to therapy, failure to return for follow-up visits, failure to meet selection criteria at entry, other protocol violations, and refusal to receive treatment, will be recorded. The potential adverse events will also be recorded despite the fact that only 0.375 J will be delivered to an acupoint.

### 2.7 Statistical analysis

All data will be presented as mean ± standard deviation. The independent *t* test and chi-square test are used to evaluate and compare the baseline patient characteristics between the experimental and sham groups. Repeated measure analysis of variance and multiple comparisons are used to evaluate the changes in the OSDI, TFBUT, Schirmer I test result, and VAS score between the experimental and sham groups. Differences are considered to be significant at a *P* value of <.05. All analyses are performed with the SPSS for Windows, version 22 (Statistics 22, SPSS, IBM Corp., Chicago, IL).

### 2.8 Data monitoring

Data monitoring committee (DMC) is not needed because laser acupuncture is general practice and noninvasive intervention.

## 3 Discussion

The prevalence of dry eye in the Taiwan population is 4.87%, and the ratio of affected women to men is 2.34.^[20]^ Two key mechanisms are thought to cause dry eye: tear hyperosmolarity and tear film instability, both of which are generally related to ocular surface inflammation.^[1]^ The therapy for dry eye traditionally involves hydrating and lubricating the ocular surface, which may provide temporary relief from the symptoms of irritation and blurred vision.^[21]^ It is important to educate the patients and make them aware that dry eye is a chronic disease that needs long-term treatment, and that evident therapeutic effects can be observed only after a certain period. Treatment for dry eye disease involves a step ladder approach that is based on the severity of the condition.^[22]^

It is thought that acupuncture might help reduce inflammation through modulation of vagus nerve activity,^[23]^ by regulating the anti-inflammatory pathways in the ocular surface or other accessory organs of the eye. The analgesic effects of acupuncture, which alleviate ocular irritation and pain, might also contribute to its therapeutic effects.^[24]^ Prior studies have shown that acupuncture can modulate the autonomic nervous and immune systems, to increase lacrimal secretion by stimulating lacrimal gland function.^[25–28]^ In comparison with traditional acupuncture for obtaining qi, laser acupuncture is not related to somatosensation but has the advantage of being noninvasive and aseptic. Laser acupuncture is also effective and needs less time than traditional acupuncture. Furthermore, there is no heat generated during the procedure that make laser acupuncture painless and safe.^[29]^ LA integrates the positive effects of both acupuncture and low-level laser therapy.^[15]^

In conclusion, laser acupuncture is expected to be effective for the treatment of dry eye in addition to conventional treatments.

## Acknowledgments

The authors thank the Biostatistics Center, Kaohsiung Chang Gung Memorial Hospital for statistics work.

## Author contributions

WLH, PCW, and LYP conceived and designed the study. PCW, CCP, and HJY recruited the participants. WLH and CCP performed the intervention. HJY and CCP collected the patients’ data. HJY and YCH interpreted and analyzed the data. WLH drafted the manuscript. HJY and YCH critically reviewed and revised the manuscript. All authors read and approved the final manuscript.

**Conceptualization:** Wen-Long Hu, Pei-Chang Wu, Li-Yen Pan.

**Data curation:** Wen-Long Hu, Pei-Chang Wu, Hun-Ju Yu, Chih-Chin Pan.

**Formal analysis:** Hun-Ju Yu, Yu-Chiang Hung.

**Investigation:** Wen-Long Hu, Chih-Chin Pan.

**Methodology:** Wen-Long Hu, Pei-Chang Wu, Li-Yen Pan, Hun-Ju Yu.

**Project administration:** Wen-Long Hu, Li-Yen Pan, Hun-Ju Yu.

**Resources:** Pei-Chang Wu, Hun-Ju Yu, Chih-Chin Pan.

**Supervision:** Wen-Long Hu, Pei-Chang Wu, Li-Yen Pan, Yu-Chiang Hung.

**Validation:** Wen-Long Hu, Pei-Chang Wu, Li-Yen Pan, Hun-Ju Yu, Chih-Chin Pan.

**Visualization:** Wen-Long Hu.

**Writing – original draft:** Wen-Long Hu.

**Writing – review and editing:** Hun-Ju Yu, Yu-Chiang Hung.
